# The Role of miR-29 Family in TGF-β Driven Fibrosis in Glaucomatous Optic Neuropathy

**DOI:** 10.3390/ijms231810216

**Published:** 2022-09-06

**Authors:** Aoife Smyth, Breedge Callaghan, Colin E. Willoughby, Colm O’Brien

**Affiliations:** 1 UCD Clinical Research Centre, Mater Misericordiae University Hospital, D07 R2WY Dublin, Ireland; 2 Genomic Medicine, Biomedical Sciences Research Institute, Ulster University, Coleraine BT52 1SA, UK

**Keywords:** glaucoma, glaucomatous optic neuropathy, fibrosis, miR-29, extracellular matrix

## Abstract

Primary open angle glaucoma (POAG), a chronic optic neuropathy, remains the leading cause of irreversible blindness worldwide. It is driven in part by the pro-fibrotic cytokine transforming growth factor beta (TGF-β) and leads to extracellular matrix remodelling at the lamina cribrosa of the optic nerve head. Despite an array of medical and surgical treatments targeting the only known modifiable risk factor, raised intraocular pressure, many patients still progress and develop significant visual field loss and eventual blindness. The search for alternative treatment strategies targeting the underlying fibrotic transformation in the optic nerve head and trabecular meshwork in glaucoma is ongoing. MicroRNAs are small non-coding RNAs known to regulate post-transcriptional gene expression. Extensive research has been undertaken to uncover the complex role of miRNAs in gene expression and miRNA dysregulation in fibrotic disease. MiR-29 is a family of miRNAs which are strongly anti-fibrotic in their effects on the TGF-β signalling pathway and the regulation of extracellular matrix production and deposition. In this review, we discuss the anti-fibrotic effects of miR-29 and the role of miR-29 in ocular pathology and in the development of glaucomatous optic neuropathy. A better understanding of the role of miR-29 in POAG may aid in developing diagnostic and therapeutic strategies in glaucoma.

## 1. Introduction

Glaucoma is the leading cause of irreversible blindness worldwide, affecting over 60 million people, a number which is predicted to rise to 118.8 million by 2040 [1]. Glaucoma refers to a group of clinical conditions rather than to an individual disorder. In general, these conditions are related by the presence of a progressive optic neuropathy. The most common form of glaucoma, primary open angle glaucoma (POAG) is characterised by raised intraocular pressure (IOP), loss of retinal ganglion cell (RGC) axons, and excavation of the optic nerve head (ONH), leading to characteristic peripheral visual field loss [2]. The key site of damage at the ONH in glaucoma is the lamina cribrosa (LC), which consists of a series of porous connective tissue plates, allowing the passage of RGC axons to exit the eye whilst maintaining structural support to the ONH. Surrounding these laminar plates are the LC cells and ONH astrocytes, which produce essential growth factors and the extracellular matrix (ECM) [3]. The laminar plates consisting of ECM components, including collagen and elastin, are subject to fibrotic remodelling, including excess deposition of elastin and collagen fibres [4,5]. Increased IOP is believed to lead to mechanical strain on the LC, disruption of these porous pathways, and resultant damage to the RGC axons, which are seen in glaucomatous injury [6].

The primary and only known modifiable risk factor in POAG is raised intra-ocular pressure (IOP). IOP is maintained by a balance between the rate of production of aqueous humour from the ciliary body and the rate of drainage of aqueous via the trabecular meshwork (TM) and the uveoscleral pathway. Resistance to outflow occurs primarily at the juxtacanalicular connective tissue due to ECM remodelling leading to raised IOP [7]. IOP-lowering treatment reduces the risk of glaucoma development and progression. Several multi-centre studies have shown that reducing IOP is neuro-protective and can delay structural and functional damage to the optic nerve axons [8]. In the Ocular Hypertension Treatment Study (OHTS), treatment reduced the risk of developing glaucoma from 9.5% to 4.4% [9]. In the Early Manifest Glaucoma Trial (EMGT) a 25% reduction in IOP resulted in less frequent and later progression compared to untreated patients [10]. However, despite adequate reduction in IOP with medical or surgical intervention, many patients still progress and develop significant visual field loss. The cumulative risk of unilateral blindness due to glaucoma is estimated to be 26.5% after 10 years and 38.1% at 20 years, while bilateral blindness has been found to occur in 5.5% of patients after 10 years and 13.5% after 20 years [11]. This suggests that the nature of POAG is multi-factorial, involving complex molecular networks and aberrant growth factor signalling^,^ working synergistically to result in the glaucomatous changes that are observed in the ONH [12]. Multiple research groups have identified transforming growth factor beta (TGF-β) as one of the major fibrotic players in glaucoma [13].

## 2. TGF-β Drives Fibrosis in Glaucoma

TGF-β is a multifunctional cytokine known to play a major role on the molecular pathways that modulate ECM in glaucomatous eyes [14,15]. The TGF-β family consists of the TGF-β1, TGF-β2, and TGF-β3 isoforms and regulates proliferation, differentiation, ECM remodelling, epithelial-mesenchymal transition (EMT), tumour progression, and apoptosis [13]. TGF-β signalling occurs via canonical (Smad dependent) and non-canonical (non-Smad dependent) pathways. The specific cellular effects of TGF-β are dependent of the isoform concentration and target tissue. Normal regulation of TGF-β is required for maintenance of tissue homeostasis; however, aberrant overexpression has been implicated in several ocular, fibrotic, and neuro-degenerative diseases. TGF-β plays a central role in fibrosis and wound healing as it induces the differentiation of fibroblasts to highly contractile myofibroblasts characterized by the enhanced expression of ECM proteins [16].

Strong evidence extrapolated from ex vivo and in vitro studies demonstrates a link between TGF-β and ocular hypertension. Raised IOP has been associated with the TGF-β-induced fibrotic response in the eye. Cultured human and animal anterior ocular models have shown that TGF-β can directly increase IOP, while animal models mimicking glaucoma suggest that elevated IOP induces physical changes at the ONH, resulting in the compression of the optic nerve, blockage of axoplasmic flow, and retinal ganglion cell death [17,18,19,20,21,22]. The LC site is considered a significant location of optic nerve fibre damage in glaucoma as LC cells are similar to myofibroblasts, which are known to be responsible for fibrotic disease development elsewhere in the human body [23] Profibrotic changes elicited by TGF-β within the ONH have been implicated in POAG and pseudoexfoliation glaucoma (PXFG) and include altered turnover of ECM components, formation of cross-linked actin networks (CLANS), upregulation of alpha smooth muscle actin (αSMA), and actin stress fibre formation [4,11,12,13,19,20,21,22,23,24].

TGF-β1 is among 183 genes upregulated in POAG LC cells compared to normal controls [22]. In eyes with POAG, over 10-fold immunoreactivity for TGF-β2 has been observed in the region of the ONH. Specifically, in the glaucomatous ONH, there is a massive increase in the levels of TGF-β2 which is mainly localized to reactive astrocytes that line the vitreous surface and occupy the prelaminar region, the compressed cribriform plates, and the nerve bundles in the lamina cribrosa [25]. In addition, exogenous treatment with TGF-β1 induces expression of ECM genes in the LC and the TM [26]. As a central mediator of myofibroblast trans-differentiation and fibrosis, TGF-β is a promising therapeutic target for glaucoma. However, clinical implementation of global TGF-β antagonism deserves cautious consideration since TGF-β exerts important immunomodulatory and tumour-suppressive effects. The complete inhibition of TGF-β as a therapeutic strategy may prove counter effective as it has multiple effects on different cells within the same organ. This highlights the need for a more targeted therapeutic strategy that can regulate the TGF-β signalling pathway at several different levels.

## 3. MicroRNAs

MicroRNAs (miRNAs) are small endogenous non-coding RNAs approximately 19–25 nucleotides in length and are located in the exons or introns of coding genes [27]. They are important negative regulators of post-transcriptional gene expression, with reports suggesting that over 60% of protein-coding genes in humans are regulated by miRNAs [28]. MiRNAs bind to partially complementary sequences within the 3′ untranslated region of target mRNAs and either induce nonsense mediated decay or translational repression. They were initially discovered in the nematode *Caenorhabditis elegans* in 1993 [29,30], and since then over 2500 miRNAs have been identified in the human genome [31].

**Figure 1:**
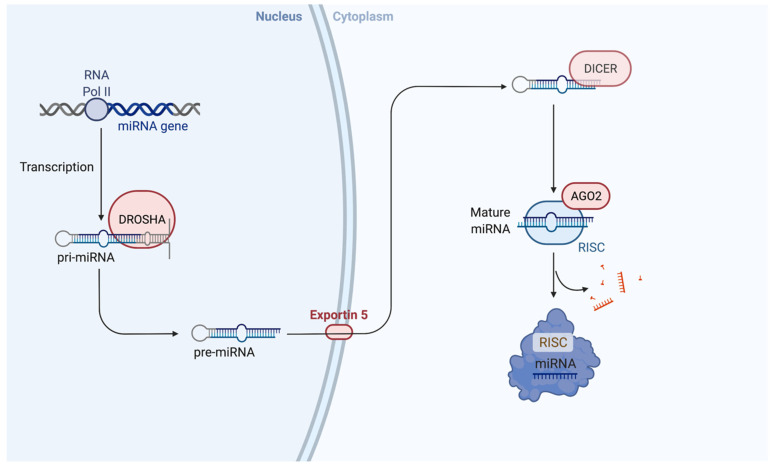
**MiRNA biogenesis.** miRNA genes are transcribed by RNA polymerase II into pri-miRNA, which are cleaved into pre-miRNA by nuclear RNase III Drosha. Pre-miRNA is transported to the cytoplasm by Exportin 5, where it is then processed into mature double-stranded miRNA by Dicer a RNase III enzyme. Finally, either strand of the mature miRNA duplex is loaded onto the Argonaut (AGO) protein to form the miRNA induced silencing complex (RISC).

Most miRNAs are transcribed by RNA polymerase II from long primary transcripts known as pri-miRNAs [32]. Only pri-miRNAs which contain the suitable stem length, a large flexible terminal loop of about 10 base pairs (bp) and the ability to produce 5′ and 3′ single-stranded RNA overhangs will be efficiently processed and mature to functional miRNAs. Pri-miRNA is cropped into the hairpin loop precursor molecule (pre-miRNAs), approximately 70 nucleotides in length, in the nucleus by nuclear RNase III Drosha [33]. This liberated hairpin structure, referred to as pre-miRNA, is transported from the nucleus to the cytoplasm via the transport receptor exportin 5 [34], where it is processed into ~22-nucleotide-long double-stranded mature miRNAs by Dicer RNAse III enzyme [35]. Pre-miRNAs often contain the sequences for multiple mature miRNAs. One strand of mature miRNA is degraded, while the other then integrates into an RNA-induced silencing complex (RISC) via loading onto the Argonaut proteins [36] (Figure 1). The uncapitalized prefix “mir-” refers to the precursor form, whereas “miR-” refers to the mature form. The suffix -3p or -3q is used to denote which end of the pre-miRNA the mature miRNA originates from.

Although some miRNAs function as a switch by repressing a single target, the majority exert their effects as modest alterations on several targets through imperfect binding which cumulatively can alter cellular phenotypes [37]. Gene regulation mediated by miRNAs occurs via two main mechanisms. Firstly, via translational repression of target mRNA by miRNAs blocking initiation of translation and/or elongation [38,39]. Secondly, miRNAs can bind to mRNAs, promoting RNA degradation through accelerated deadenylation and decapping [40,41].

MiRNAs play critical roles in multiple physiological and pathological processes. Due to their unique expression patterns and their ability to target numerous transcripts often in the same biological process, miRNAs can potentially regulate the expression of many genes in a tissue- or cell-specific manner. They operate in complex networks in which one mRNA can be regulated by multiple miRs and one miR can regulate multiple genes targets [42]. An entire signalling pathway can be regulated by miRNAs in physiological and disease processes. The dysregulation of miRNAs in disease states can be used to develop biomarkers [43] and provide insight into the underlying pathophysiological basis of the disease [44]. Abnormally expressed miRNAs have been identified in cancer, fibrosis, Alzheimer’s, and cardiovascular disease [45,46,47,48]. In POAG, many miRNAs have been detected in patient-derived samples [49] and play a role in the TM [50], retina [51], and ONH [52].

## 4. MicroRNAs That Regulate TGF-β Signalling

By targeting up- or downstream signalling molecules in the TGF-β signalling pathways, miRNAs can promote or inhibit the development of fibrosis. MiRNA-based manipulation of the TGF-β signalling pathway is a new approach to target fibrosis [53] and numerous studies have been published detailing the reciprocal crosstalk between miRNAs and TGF-β [54,55,56,57]. Both in-silico and experimental validation analysis has shown that miRNAs can influence TGF-β signalling at several transcriptional and post-transcriptional levels. Nearly all the TGF-β members involved in the canonical signalling pathway have been shown to be influenced by miRNAs. Reports have also demonstrated the regulation of non-canonical TGF-β/PI3K/AKT signalling pathways by miRNAs, supporting their potential role in the diagnostic and therapeutic management of fibrotic diseases [54]. Interestingly, TGF-β signalling itself can enhance the maturation of miRNAs by binding to a component of the Drosha complex (p68) and initiating a bidirectional functional loop [57,58,59].

TGF-β induced fibrosis related miRNAs has been studied more extensively in other tissues, such as the lung, liver, kidney, heart, and skin [60,61]. In cardiac fibrosis, miR-92a regulates the inhibitory Smad7, promoting the fibrotic process while miR-26a has been shown to directly target Smad1 [62]. The miR-200 family targets TGF-βR1 and SMAD2; therefore, downregulation of the miR-200 family results in increased EMT [59]. In the kidney, miR-29 can inhibit disintegrin metalloproteases (ADAMs) involved in TGF-β signalling, reducing collagen expression and the subsequent development of renal fibrosis [63]. In the eye, many miRNAs have been found to be abundantly expressed and play important roles in ocular physiology and pathology. These include miR-200 [64] miR-184 [65] miR-29 family [66] and miR-21 [67]. Expression profiling of miRNAs in the anterior segment of healthy donors including cornea, TM, and ciliary body samples reported 378 miRs collectively expressed [68].

## 5. MiR-29 Family

**Figure 2:**
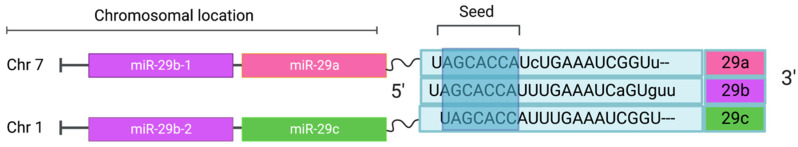
**Schematic representation of the miR-29 family members: miR-29a, -29b, and -29c.** The family is transcribed from two chromosomal positions forming a bi-cistronic transcriptional unit; both miR-29a and miR-29b1 are found on chr7, while miR-29b2 and miR-29c are located on chr1. miR-29 family members have identical seed sequences (darker blue box) along with similar mature miRNA sequences.

The microRNA-29 (miR-29) family, which this review will discuss in more detail, includes miR-29a, miR-29b-1, miR-29b-2, and miR-29c. MiR-29b-1 and miR-29b-2 have identical mature sequences and are therefore collectively known as miR-29b. MiR-29a and -29b-1 are encoded on chromosome 7q32.3, while the miR-29b-2 and -29c cluster are found on chromosome 1q32.2 (Figure 2). All four members have a common seed sequence in nucleotides 2 to 8 and largely regulate a similar group of target mRNAs [69]. Mir-29, like other miRNAs, is transcribed by RNA polymerase II [70]. MiR-29a and -29b-1 are transcribed as a primary transcript unit [71].

MIR-29 expression is regulated at both a transcriptional and post transcriptional level. Multiple transcriptional factor binding sites on miR-29 have been identified, including a Smad3 binding site in miR-29b-2 [72], a myc binding site in both the miR-29b-1/a and -29-2/c clusters [73] and three NF-kB binding sites in miR-29-1/a region [74]. Similarly, post transcriptional regulation of miR-29 has been reported in anaplastic large cell lymphoma, where t(6;7)(p25.3;q32.3) spontaneous translocation was associated with upregulation of miR-29 on 7q32.3 [75].

Although each miR-29 family member shares the common 7-nt seed sequence, unique sequence features have been reported. One notable difference is that miR- 29b, unlike -29a or c, has a distinct hexanucleotide terminal motif required for nuclear localization [76]. In exogenous delivery of miR-29b, mutations in this region impair localization to the nucleus [77]. Deletion of this hexanucleotide motif led to higher cytoplasmic localization of miR-29b and enhanced effects with downregulation of its target, knowledge of which may be beneficial in enhancing miR-29b therapeutic effects [78]. MiR-29a is mainly localized to the cytoplasm and has a distinct cytosine residue at position 10 [77]. Deep sequencing analysis found -29c to be enriched to a greater extent in the nucleus than the cytoplasm [79]. Alternatively, miR-29b and -29c contain a tri-uracil sequence at position 9–11, responsible for rapid decay. Replacement with the cytosine residue in -29a leads to greater stability [80].

The miR-29 family have been implicated in many cellular functions, including apoptosis [81,82], proliferation, and differentiation [83], and play an essential role in development and normal physiology. MiR-29 knockout (KO) mice demonstrate developmental defects and growth retardation [84]. Downregulation of miR-29 has also been linked to the development of many cancers, including lung [85], colon [86], liver [87], and acute myeloid leukaemia [88]. Tumour suppressor effects of miR-29b were demonstrated in xenograft leukaemia models and exogenous delivery of miR-29b induced apoptosis of tumour cells and dramatic reduction in tumorigenicity [88,89]. In contradiction to this, other reports propose that miR-29 is an oncogene in gastric and pancreatic cancers [73], strengthening the argument that the biological function of miRNAs is cell-type- and organ-specific.

## 6. MiR-29 in Fibrosis

The miR-29 family are key players in fibrotic activity in multiple organs including the heart, lungs and kidney. Each family member targets many different genes encoding proteins essential for both the physiological and pathological formation of ECM including collagen, laminin, fibrillin, and elastin [90,91]. The miR-29 family is unique in that in targets 20 collagen mRNA species, with no other reported miRNA targeting more than 11 collagen transcripts, proposing it is a “master regulator” of fibrosis or the “master fibromiRNA” [46,92].

MiR-29a has been found to be a negative regulator of TAB1, a gene responsible for the regulation of inflammation. An increase in TAB1 leads to elevated expression of tissue inhibitor of matrix metalloproteinase-1 (TIMP-1), a widespread inhibitor of MMPs, demonstrating its role in fibrosis [93]. Interestingly, the expression levels of miR-29 differ with organ-specific cell types. MiR-29 levels were 100 times higher in hepatic stellate cells (HSCs) than hepatocytes [94] Similarly, higher levels were reported in cardiac fibroblasts than in myocytes [95]. Both studies demonstrate high expression levels in cell types that play a role in the regulation of ECM.

The suppression of miR-29 is strongly linked to the pro-fibrotic TGF-β. By binding to TGF-β receptors, TGF-β1 phosphorylates Smad2 and Smad3 leading to nuclear translocation and regulation of the expression of approximately 60 target ECM genes [63]. MiR-29 is a known downstream target in the TGF-β/Smad pathway [96], and Smad3 signalling results in downregulation of miR-29 [97].

The antifibrotic role of miR-29 has been extensively investigated in pulmonary fibrosis [93]. In both bleomycin-induced fibrotic lung disease and idiopathic pulmonary fibrosis, miR-29 family member levels were significantly reduced [96,98]. MiR29 expression levels are inversely correlated with the severity of pulmonary fibrosis [92]. TGF-β stimulation reduced miR-29a-c levels further and upregulated collagen I, III and fibronectin in the fibrotic lung. This downregulation of miR-29 was prevented in Smad3 knockout mice, indicating regulation of miR-29 via the Smad3 signalling pathway. Similarly, knockdown of miR-29 in human foetal lung fibroblasts correlated with an increase in profibrotic genes including ADAM12 and ADAMTS9 [99]. In IMR90 cells, TGF-β1 induced COL1a1 expression was blocked by miR-29 mimics for each family member. The overexpression of miR-29 using specific mimics for miR-29a, b and c family members demonstrated a disruption to PI3K/AKT phosphorylation and resulted in atypical collagen expression, suggesting targeting of the PI3k-Akt pathway [100]. In a model of liver fibrosis, all three members of the miR-29 family were downregulated [101]. MiR-29b is also reduced in HSCs, a primary producer of collagen in the liver. Transfection of HSCs with miR-29b significantly attenuated the expression of Col1a1 and Col1a2 [102]. MiR-29b has been reported to be the most effective suppressor of type 1 collagen in HSCs among several miRNA whose expression levels were under the control of TGF-β1 [100]. In addition, over expression of miR-29b in rat HSCs inhibited lysyl oxidase and heat shock protein 47, two essential proteins in ECM maturation [103].

The anti-fibrotic reports of miR-29 are also evident in cardiac pathophysiology. MiR-29c expression was decreased in a model of cardiac hypertrophy in mice [104]. Following acute myocardial infarction in mice, all mir-29 family member levels were downregulated in cardiac tissue adjacent to the infarct compared to remotely isolated tissue from the same donor [92]. In congestive heart failure, miR-29 expression was reduced in atrial fibroblasts with an associated increase in ECM target genes including fibrillin and COL1A1 and COL3A1 [105]. In contrast to its protective antifibrotic effects in myocardial cells, one report states miR-29a and miR-29c overexpression promoted apoptosis, whereas downregulation protected against myocardial ischaemia–reperfusion injury [106]. The pro-apoptotic effects of miR-29 have been reported in several cell type [107,108,109]. MiR-29 has been shown to activate p53 via p85α and CDC42 and therefore promote apoptosis [108,109]. However, under mild stress conditions, p53 can function as an antioxidant and promote cell survival [110]. Thus, the pro-apoptotic effects of miR-29 through p53 signalling may be dependent on the specific cell type and stress level.

The TGF-β/Smad signalling pathway is a well-known regulator of renal fibrosis. In renal tubular epithelial cells, TGF-β1 negatively regulates miR-29b expression via Smad3. Induction of renal fibrosis by unilateral ureteric obstruction in mice induced a significant reduction in miR-29a,b, and c [63]. As reported in pulmonary fibrosis, expression of miR-29 was significantly upregulated in Smad3 knockout mice, with subsequent inhibition of renal fibrosis [72]. In diabetic nephropathy, miR-29b was downregulated in mesangial cells via TGF-β/Smad mechanisms [111]. Loss of miR-29b led to progression of microalbuminuria and renal fibrosis. In systemic sclerosis (SS), a multisystem fibrotic disorder, miR-29a was strongly downregulated compared to healthy controls. TGF-β, PDGF-B, and IL-4 also reduced miR-29a levels in controls similar to levels seen in SS fibroblasts [112,113]. In keloid vs. healthy fibroblasts, miR-29a–c levels were significantly reduced—miR-29b expression in particular. In keloid fibroblasts transfected with pre-miR-29a, type I and III collagen mRNA and protein levels were decreased [114].

It is clear that miR-29 plays a crucial role in fibrosis in many organs via regulation of multiple signalling pathways in addition to direct inhibition of ECM proteins. Novel research on miR-29 as a therapeutic strategy in fibrosis is ongoing. Exogenous miR-29b gene delivery following bleomycin administration in normal lung tissue prevented the development of pulmonary fibrosis [96]. In diabetic nephropathy, ultrasound assisted gene therapy restored miR-29b expression levels thus attenuating fibrotic changes [111]. Multiple studies have shown that the level of fibrotic transformation in varying pathological processes can be regulated by targeting the TGF-β driven downregulation of the miR-29 family in specific tissues [113,114,115].

## 7. MiR-29 Family in Eye Disease

The majority of research on miR-29 in ocular pathology focuses on its role in glaucoma. However, it has also been reported in several anterior [66] and posterior segment conditions [116]. In Fuchs endothelial corneal dystrophy (FECD), a common condition resulting in disruption of the corneal endothelium [28], the differential expression of 87 significant miRNAs was analysed. The miR-29 family was the most significantly downregulated in the endothelial tissue from FECD patients. Transcriptional overexpression of the miR-29 target genes, COL1A1 and COL4A1, was noted in the same samples. Interestingly, given its role in miR-29 downregulation, TGFβ-1 has been reported to be upregulated in FECD samples [117]. In another study, treatment of corneal endothelial cells in FECD with miR-29b led to a significant drop in expression levels of ECM genes, including COL1A1, COL4A1, and LAMC1 [118]. In a guinea pig model of myopia, miR-29a,b, and c expression was increased in the sclera and the expression levels of Col1a1 was reduced [119]. The differential expression of the miR-29 family was also investigated in diabetic retinopathy models [115] and these studies proposed that miR-29 may play a protective role in the regulation of retinal pigment epithelium (RPE) apoptosis induced by hyperglycaemia [120].

## 8. MiR-29 Family in Glaucoma

Given the well-reported role of miR-29 in ECM production and fibrotic transformation in various tissues, it is not surprising that research on miR-29 in ocular pathology focuses mainly on its role in the development of fibrosis at the TM and ONH in POAG. MiR-29a, 29b, and 29c are expressed in TM cells [121] and lamina cribrosa cells [52]. In aqueous humour samples from healthy and glaucomatous human donors, miR-29a, b, and c were detected and are among the most prevalent miRNAs isolated from glaucomatous samples [122,123]. In array studies, miR-29b was downregulated in Tenon’s ocular fibroblasts (TF) stimulated with TGF-β1. TGF-β2 also reduced miR-29b expression in both normal and glaucomatous TF cells in vitro [124]. Overexpressing miR-29b in TF cells also inhibited the expression of COL1a1, SP1, and PI3Kp85a, supporting the role of miR-29b in the regulation of PI3K signalling in fibrosis and subsequent collagen production [125]. In vivo, miR-29 was found to be downregulated in the retina of a CNS-injury-induced model of glaucoma [126]. Contradictory to other reports, Liu et al. demonstrated reduced miR-29b expression in the retinal tissues of glaucoma rats vs. control [127]. In addition, they suggested that miR-29b may induce apoptosis in human TM cells and silencing of miR-29 protected HTM cells against oxidative injury via ERK pathway.

ECM remodelling of the LC is a key process in the development of glaucomatous optic neuropathy. There is limited published research on miR-29 specifically at the lamina. However, an important paper by Lopez et al. found that miR-29a and miR-29c expression levels were downregulated in glaucomatous LC cells compared to age-matched controls. They also found miR-29b was downregulated in glaucoma samples. However, this was not statistically significant [52]. Interestingly, treatment of LC cells with TGF-β2 resulted in an additional reduction in miR-29c expression. LC cells were subsequently transfected with miR-29c, which resulted in the downregulation of type I and IV collagen, suggesting that TGF-β2 and miR-29 regulate ECM synthesis in the LC cell [52]. Restoration of miR-29 expression levels at the ONH may aid in the regulation of TGF-β-induced ECM overproduction.

Induction of oxidative stress in cultured human TM cells resulted in the downregulation of miR-29b and increased expression of ECM genes [128]. Transfection of these cells with a miR-29b mimic resulted in reduced expression of an array of collagen genes (COL1A1, COL1A2, COL4A1, COL5A1, COL5A2, COL3A1) and ADAM12, a profibrotic gene which was also identified as a direct target of miR-29b. This suggests that miR-29b may be able to prevent excess ECM deposition in TM and modulate changes in IOP due to outflow restriction [128]. Further research from Luna et al. demonstrated that TGF-β2, but not TGF-β1, downregulated all three members of the miR-29 family in TM cells [67]. However, Villarreal et al. found that TM cells treated with TGF-β2 induced miR-29a expression and miR-29b suppression [121]. Additionally, in TGF-β2-treated TM cells, up-regulation of miR-29 reduced the expression of previously upregulated ECM proteins. SMAD3 modulates miR-29b expression via the TGF-β signalling pathway under both basal and TGF-β2 conditions in the cultured TM cells [121].

The canonical Wnt signalling pathway is also crucial in the regulation of ECM expression and aqueous outflow [129]. Lithium chloride (LiCl) stimulation, a known activator of β-catenin, upregulated miR-29b levels, but no significant change was observed in miR-29a or c expression in primary TM cells [129]. These studies suggest that miR-29b expression is modulated by a variety of factors, including LiCl, TGF-β, and oxidative stress, and that miR-29b plays a central role in ECM synthesis. MiR-29b is regulated by the TGF-β signalling pathway and the canonical Wnt signalling pathway, both essential ECM pathways. Evidence of cross-inhibition between the TGF-β/SMAD and canonical Wnt pathways in the TM has been reported [130]. Interestingly, miR-29a was also shown to be upregulated in TM cells following mechanical stretch [131]. The crosstalk between different signalling pathways plays a role in ocular fibrosis associated with glaucoma.

Although targeting fibrotic transformation at the TM is beneficial for aqueous outflow and IOP modulation, targeting fibrosis of the LC may crucially alter the common endpoint of glaucoma, irreversible ONH damage. Despite the limited amount of research published on miR-29 at the ONH in glaucoma, available evidence demonstrates the importance of miR-29 in TGF-β-induced fibrosis at the ONH in POAG. TGF-β2 expression levels are elevated in LC cells isolated from glaucoma patients compared to age-matched controls [22]. Our research group has previously demonstrated that the LC cells are sensitive to mechanical stress/strain, and in response they upregulate the expression of ECM genes and TGF-β [26]. Upregulation of TGF-β and ECM genes leads to increased collagen deposition and the disorganization of collagen and elastin fibres, resulting in destruction of the normal porous pathways of LC and RGC axon degeneration [4,5]. Previously, we have shown that TGF-β upregulated collagen expression at the LC [26]. As demonstrated by Lopez et al., induction of ECM proteins by TGF-β2 is attenuated by miR-29c, indicating miR-29c may also regulate TGF-β2 signalling. Together, these results support the concept that miR-29 can influence the TGF-β2 signalling pathway in ECM synthesis in LC cells [54]. Further research is required to expand on the role of miR-29 in the development of glaucomatous optic neuropathy. Investigating whether TGF-β inhibits the transcription of miR-29 or affects the processing of this microRNA would be a beneficial next step.

There is increasing evidence of the role of miR-29 in the development of fibrosis in POAG. Future research on the development of miRNA therapies to control ECM production may offer a novel disease-modifying therapeutic approach in POAG. Multiple papers investigate the possibility of restoring downregulated miR-29 levels either through transfection with mimics or the use of viral vectors. Subconjunctival injection of lentivirus-mediated miR-29b following glaucoma filtration surgery in rabbits found reduced collagen 1a1 expression and fibroblast numbers at post-operative day 28, resulting in lower IOP and sustained bleb function compared to controls [132]. Interestingly, it was noted that overexpression of miR-29b provided protection to subconjunctival tissue against collagen production and fibrosis, suggesting it may play a role in attenuating bleb scarring following glaucoma filtration surgery [124]. In a study examining corneal and retinal fibrosis, an oligonucleotide mimic of miR-29b, MRG-201, was administered topically to both a rat cornea post-alkali burn and via intravitreal injection to a rabbit model of proliferative vitreoretinopathy. MRG-201 was found to reduce collagen expression and inhibit corneal and retinal fibrosis [133].

## 9. Conclusions

Understanding the role of miRNAs in health and disease in the eye may prove central to dissecting the physiological and pathophysiological mechanisms impacting glaucoma. The ability of miRNA manipulations to alter gene expression in vitro and in vivo has raised the possibility of miRNA-based therapeutics [112,133]. Several miRNA therapeutics are undergoing evaluation in preclinical and clinical studies, including miRNAs which target fibrotic responses in the lung, liver, heart, and kidney [133,134,135,136]. There are several methods of manipulating miRNA expression, including miR mimics, anti-miRs, target site blockers, and miRNA sponges [133]. The anterior segment and TM offer many advantages for miRNA-based therapies, including accessibility and non-invasive observation of the phenotypic effects of therapy in vivo. Several potential delivery options are feasible [136,137]. Several challenges must be overcome, and understanding the miRNA–mRNA interactome is critical to development miRNA therapeutics for fibrosis in the LC and TM.

## Data Availability

Not applicable.
